# Impacts of nucleotide fixation during soybean domestication and improvement

**DOI:** 10.1186/s12870-015-0463-z

**Published:** 2015-03-08

**Authors:** Shancen Zhao, Fengya Zheng, Weiming He, Haiyang Wu, Shengkai Pan, Hon-Ming Lam

**Affiliations:** Centre for Soybean Research, Partner State Key Laboratory of Agrobiotechnology, The Chinese University of Hong Kong, Shatin, New Territories, Hong Kong; BGI-Shenzhen, Main Building, Beishan Industrial Zone, Yantian District, Shenzhen, 518083 China

**Keywords:** Soybean domestication, Genetic improvement, Artificial selection, Nucleotide fixation, Plant-pathogen interaction

## Abstract

**Background:**

Plant domestication involves complex morphological and physiological modification of wild species to meet human needs. Artificial selection during soybean domestication and improvement results in substantial phenotypic divergence between wild and cultivated soybeans. Strong selective pressure on beneficial phenotypes could cause nucleotide fixations in the founder population of soybean cultivars in quite a short time.

**Results:**

Analysis of available sequencing accessions estimates that ~5.3 million single nucleotide variations reach saturation in cultivars, and then ~9.8 million in soybean germplasm. Selective sweeps defined by loss of genetic diversity reveal 2,255 and 1,051 genes were involved in domestication and subsequent improvement, respectively. Both processes introduced ~0.1 million nucleotide fixations, which contributed to the divergence of wild and cultivated soybeans. Meta-analysis of reported quantitative trait loci (QTL) and selective signals with nucleotide fixation identifies a series of putative candidate genes responsible for 13 agronomically important traits. Nucleotide fixation mediated by artificial selection affected diverse molecular functions and biological reactions that associated with soybean morphological and physiological changes. Of them, plant-pathogen interactions are of particular relevance as selective nucleotide fixations happened in disease resistance genes, cyclic nucleotide-gated ion channels and terpene synthases.

**Conclusions:**

Our analysis provides insights into the impacts of nucleotide fixation during soybean domestication and improvement, which would facilitate future QTL mapping and molecular breeding practice.

**Electronic supplementary material:**

The online version of this article (doi:10.1186/s12870-015-0463-z) contains supplementary material, which is available to authorized users.

## Background

The cultivated soybean [*Glycine max* (L.) Merr.] is an economically important crop that grown all over the world. With an average of ~38% protein and ~18% oil content in seeds, soybean provides 69% of dietary protein and 30% of vegetable oil consumption worldwide (www.usda.gov). Modern soybean cultivars were originally domesticated from its wild progenitor (*Glycine soja* Sieb. & Zucc.) more than 3000 years ago, which was an endemic species in China [1]. Since then, a variety of morphological and physiological changes except for reproductive isolation have occurred that distinguish soybean cultivars from their wild ancestor. Wild soybeans possess much higher adaptability to various natural environments such as drought and salt stress, whereas cultivated soybeans exhibit a bush-type growth habit with large seeds, variable seed coat colors and a stout primary stem. Wild soybeans also differ in the extent of photosynthesis capacity, pod dehiscence and number from cultivated soybeans [2-4].

Heritable changes occurred during plant domestication are being revealed by gene mapping and genomic analyses [5]. The availability of soybean genome and high throughput sequencing technologies provides excellent opportunity to excavate the domestication events and phenotypic diversification at the genome level [6]. Re-sequenced soybeans representing wild and cultivated accessions revealed the nature and extent of genetic diversity in both populations [7-9]. Another research reported a reservoir of genes that were affected by early domestication and modern genetic improvement [10]. Besides, several domestication-related traits have been studied and proposed to be controlled by a small number of genes or several major QTLs [11,12]. However, more analyses are needed to delimit the regions of these QTLs and the footprints of domestication for further gene mapping.

From an evolutionary perspective, if a mutation happens to be beneficial to the species, it will spread to the population immediately by selection [13]. During crop domestication, strong selective pressure caused traits of interests to be fixed in a founder population in quite a short time [14]. Probably, advantageous mutations underlying traits of interests will be subject to fixation in the population. These fixation events differ from those in natural populations, because artificial selection usually acted on alleles that were likely neutral or nearly neutral before domestication. Thus, understanding nucleotide fixation driven by artificial selection is indispensable to complete the picture of soybean evolution. In this research, the published soybean sequencing data were collected to identify single nucleotide variations (SNVs), based on which we detected the genomic regions affected by artificial selection during domestication and improvement. In these footprints, nucleotide fixations that happened in all cultivars were potentially caused by artificial selection, and the genes with these nucleotides were further analyzed, and some of these genes were associated with agronomic traits through functional annotation and QTL meta-analysis. This kind of investigation will provide clues to understand the differentiation of wild and cultivated soybeans. Besides, fundamental practical information will be obtained for future enhancement of cultivars through traditional breeding and transgenic methods.

## Results

### Estimation of single nucleotide variations among soybean populations

Recently, a set of diverse soybean individuals was sequenced and reported based on NGS platforms [7,8,10]. These soybeans, representing wild and cultivars that mainly consist of landrace and modern elite accessions in East Asia, were selected based on intensive molecular and phenotypic analysis to maximally reflect the genetic diversity of soybeans (Additional file 1: Table S1). It provides us an important resource to depict the genetic diversity of wild and cultivated populations, and to detect the footprints of domestication events. Thus, we downloaded all the short reads of sequencing soybeans from NCBI Short Read Archive under accession numbers SRA020131, SRA009252, SRP015830, and ERP002622. These reads were aligned to the soybean reference genome *Glycine max* (Phytozome v9) with SOAP2 [15], and were subsequently used to detect SNVs with SOAPsnp pipeline [16]. A total of 9,820,934 SNVs were identified across all accessions, of which 8,168,883 and 5,201,747 appear in wild and cultivars, respectively. Previous reports with the same pipeline have shown that the SNV calling accuracy is 95-99%, with false-positive and false-negative rates to be ~2% and ~3%, respectively [17-19].

**Figure 1:**
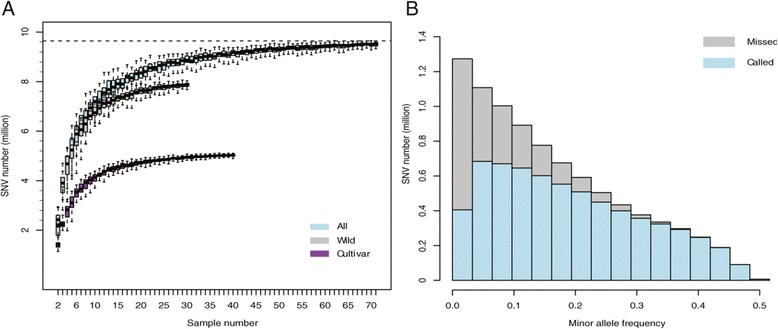
**Detection of single nucleotide variations in sequencing soybean accessions. (A)** Accumulated SNV coverage in cultivars, wild and all accessions; **(B)** Distribution of missing SNVs in previous report by Lam et al.

To estimate the coverage of these SNVs in the whole soybean germplasm, we employed a random sampling approach to investigate the accumulation of SNVs detected in different accessions (Figure 1A). The end of the SNV curve tends to be flat, which indicates that the SNVs identified here probably reach saturation in soybean germplasm. It is sufficient for as few as 48 accessions to detect 95% of all SNVs in different populations. For cultivated soybeans, only 30 individuals can achieve 95% of SNVs. Approximately 5.2 million SNVs would reach saturation in cultivars, which are far less than those in wild soybeans. In previous work [7], Lam *et al* reported 6.3 million SNVs in 31 soybeans, while we discovered 2,481,645 more in the same individuals by a larger population. A large number of rare SNVs and those with low allele frequency were missed in former analysis due to strict filtering conditions and a small number of individuals (Figure 1B). Although some very rare SNVs still remain to be discovered, we have identified a substantial majority of the common SNVs in soybeans.

**Figure 2:**
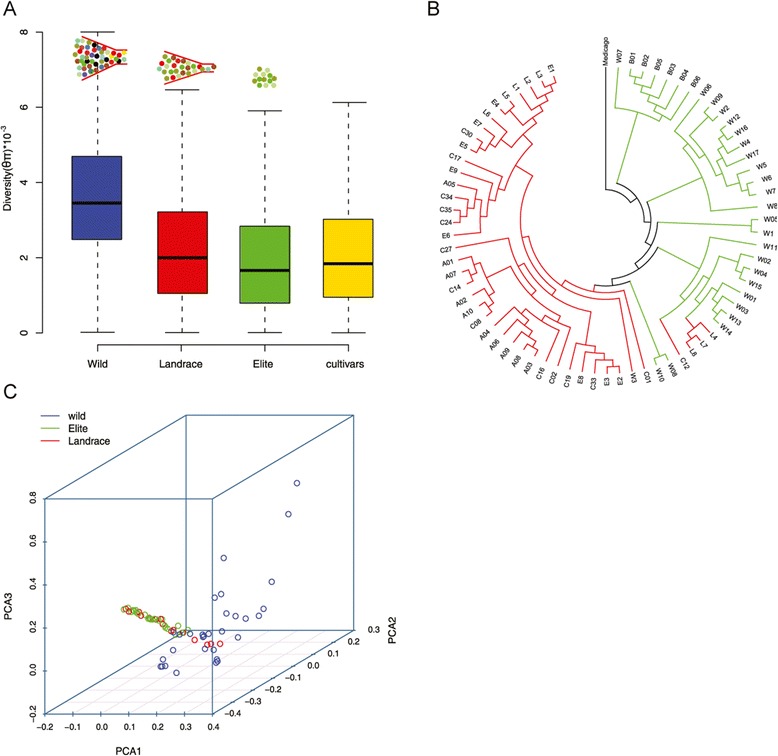
**Analysis of genetic diversity and phylogenetic relationship among soybean accessions. (A)** Reduction of genetic diversity from wild, to landrace and then to elite soybeans; **(B)** A neighbor-joining tree; **(C)** Principal component analysis of soybeans.

Soybean has suffered several genetic bottlenecks, such as early domestication producing lots of Asian landrace, the introduction of few landraces to North America, and modern extensive breeding activities [20]. Subsequently, different level of genetic diversity was reduced during these human-mediated events. More SNVs were identified in wild than in cultivated accessions. Two common statistics used to measure nucleotide diversity are the pairwise divergence per nucleotide *θ*_*π*_ [21] and Watterson estimator *θ*_*w*_ [22] that corrected for sample size. Whole-genome analysis using these parameters shows a higher level of genetic diversity in wild populations (Figure 2A). Estimated by *θ*_*π*_, the average diversity within wild, landrace and elite cultivars are 3.84 × 10^-3^, 2.40 × 10^-3^, and 2.08 × 10^-3^ per nucleotide, respectively. Considering the cultivars consist of landrace and elites, the average *θ*_*π*_ is 2.25× 10^-3^ in cultivated population. It is notable that the cultivars have retained only 58.6% of the sequence diversity present in wild soybeans, which is lower than previous estimation [7,20]. The genetic diversity was reduced by 37.5% in early domestication and further reduced by 8.3% in genetic improvement.

The reduction of genetic diversity eroded by artificial selection could also be reflected by phylogenetic tree (Figure 2B) and principle component analysis (PCA, Figure 2C). The wild soybeans shattered in a loose 3-dimension space, while cultivated soybeans formed a relatively tight cluster distinct from the wild individuals. Within the cluster, however, the landraces were not clearly separated from elite cultivars. Some landraces mixed with wild group in our analysis, indicating the early domestication process probably accompanied with considerable gene flow with the wild ancestors. In addition to artificial selection, the genetic erosion can also reflect the narrow genetic base of cultivated soybeans [23]. Analysis of representative wild and cultivated soybeans provides us a comprehensive insight into such evolutionary events that affected population dynamics of soybeans.

### Detecting artificial selection and nucleotide fixation in soybeans

**Figure 3:**
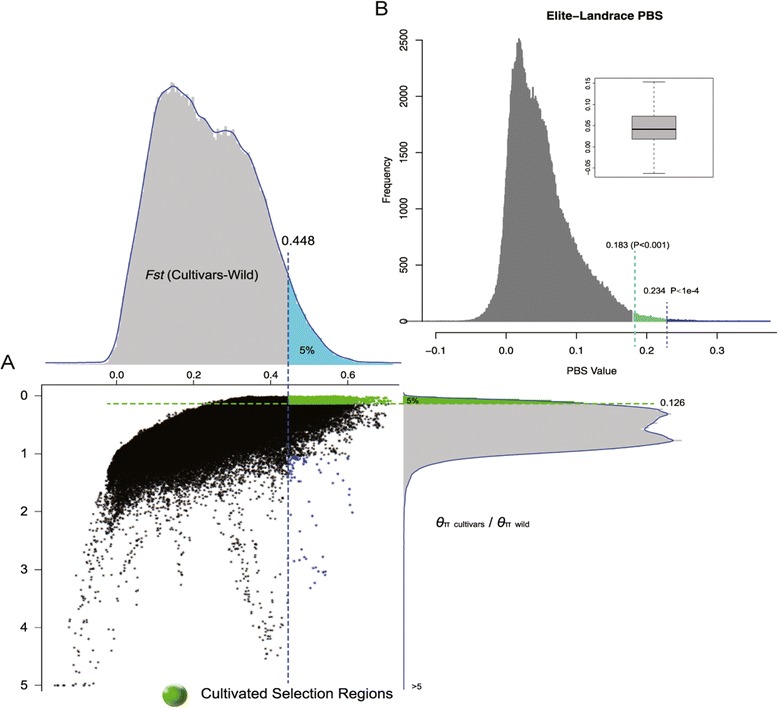
**Footprints of artificial selection during (A) early domestication and (B) modern improvement.**

The signal of artificial selection could be detected by the loss of genetic diversity, which shaped selective sweeps around beneficial alleles on the genomes [24-26]. To further elucidate the effects of domestication, we detected the genomic regions showing artificial selection signals by genetic bottleneck model [18,19] and population branch statistics [27]. The sequenced accessions except C12 and C16 were grouped into wild and cultivated population to detect selection signals in early domestication process. Using a sliding window approach, we calculated the distribution of *θ*_*π*_ and Tajima’s *D* [28] in wild and cultivated populations along the genome. Regions with significantly lower *θ*_*π*_ (*Z* test, *P* < 0.05) and lower Tajima’s *D* (*Z* test, *P* < 0.05) in cultivars than that in wild accessions were treated as putative candidates that were affected by early domestication (Figure 3A). However, signals of very recent natural selection could be easily omitted using the above bottleneck model. To detect signatures that shaped in modern crop improvement, we employed an effective method known as population branch statistics. Taking wild soybeans as control, we recalculated the divergence index *F*_*st*_ [29] in a sliding window along the genome, based on which we detected significant signals (*P* < 0.001 after Bonferroni correction) to infer selective footprints from landraces to elite cultivars (Figure 3B). This approach had been shown to be effective in identifying recent artificial selection considering the very short time of modern breeding practice [18]. A total of 598 regions comprising 27.9 Mb genome sequences and 286 regions with a length of 12.7 Mb were affected by early domestication and genetic improvement, respectively. Based on the latest annotation, 2,255 genes with 3,100 transcripts were involved in early domestication, whereas 1,051 genes with 1,462 transcripts were affected in subsequent improvement.

During the human-mediated breeding process, the strongly selected advantageous mutations could become fixed as these mutations increase in frequency in a population [11,13]. A selective sweep is shaped when a selected mutation goes to fixation, because it reduces variability in the neighboring region where neutral variants are segregating [30,31]. A nucleotide fixation locus was defined when a SNV has a unique genotype in one population while it exhibits polymorphic genotypes in the others. To better understand how genes were affected by domestication events, we primarily focused on those with nucleotide fixation in the selective footprints. We calculated the likelihood of genotypes of each individual and then we allocated the allele type with the maximum likelihood back to each individual as the consensus genotype. After calibration, 101,292 nucleotide fixations were identified in the selective regions in cultivars, which could be potentially caused by artificial selection.

**Figure 4:**
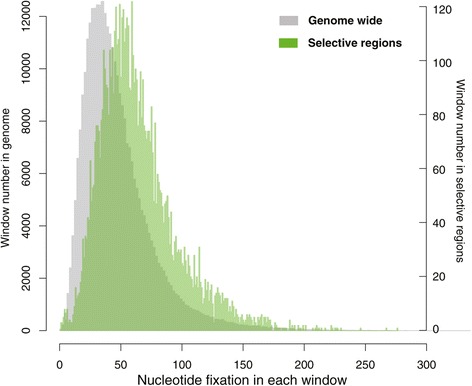
**The distribution of nucleotide fixation over the genome versus in the selective regions.** The window size was set to be 20 kb.

Compared with the genome-wide distribution, nucleotide fixations happened more frequently in the candidate regions of artificial selection (Figure 4). Nucleotide fixation accumulated substantially in cultivars and happened unevenly along chromosomes (Additional file 2: Figure S1), indicating that some chromosomes were more susceptible to be affected by artificial selection. Nucleotide fixation also explains the reduction of genetic diversity in cultivated crops compared with their wild ancestors. We analyzed the allele frequency of SNVs in wild soybeans that were fixed in cultivars, as it represents the initial status of these nucleotide fixations before domestication. The frequency spectrum shows that these SNVs were almost neutral at the beginning of domestication (Additional file 3: Figure S2). Since non-synonymous substitutions may result in a change in functions, they are subject to natural or artificial selection [32]. Of the nucleotide fixation happened in early domestication, 24,316 located in coding sequences and 2,162 of them caused non-synonymous substitutions in 1,188 genes, which altered the amino acid sequences of the proteins. For those loci fixed in modern improvement, 8,065 located in coding sequences with 756 non-synonymous in 489 genes. Apparently, more nucleotide fixations were introduced to cultivars during domestication than those during improvement.

A central question in analyzing the genetic variations in a given population is to explore whether the population has different substructures [29,33]. When analyzing the nucleotide fixations by PCA and phylogenetic tree, two distinct clusters shaped between the cultivars and wild soybeans (Additional file 4: Figure S3). Some noise always exists in inferring phylogenetic relationships among individuals, especially when they are subject to introgressive hybridization [34,35]. Cultivars tightly joined together without noise, supporting the hypothesis of a single rather than multiple evolutionary origins in soybean domestication [36,37].

### Nucleotide fixation in wild soybeans

In the process of nucleotide substitution, the fixation of a mutation could spread through the population by random genetic drift or extreme natural selection [38]. In the regions affected by artificial selection, 4,111 nucleotide fixations happened in wild soybeans, which located in 875 transcripts corresponding to 723 genes. Nucleotide fixation happened more frequently in cultivars compared with wild soybeans. To some degree, artificial selection could have promoted the occurrence of fixation events. However, genetic bottlenecks caused by domestication often results in a smaller effective population size of cultivars than that of wild soybeans, which would also contribute to an elevated level of nucleotide fixation. Genes affected by nucleotide fixations were involved in kinds of biological activities as described in the Kyoto Encyclopedia of Genes and Genomes (KEGG) database (Additional file 5: Figure S4).

The ability of resistance to pathogen in wild soybeans is much broader than that in cultivated soybeans [23,39]. Interestingly, *Glyma20g08290* gene is an ortholog of the disease resistance gene *RPM1*, which was detected and characterized using molecular genetic approach in Arabidopsis [40]. In soybeans, the *RPM1* gene was recently reported being under purifying selection [41]. It serves as an example that natural selection in the wild population also caused nucleotide fixations, although its strength was less than artificial selection.

### Agronomic traits affected by selective nucleotide fixation

During domestication, artificial selection is thought to have extremely strong selective pressure on ancestral population for desired phenotypes [42]. The strong selection exerted by human led to an excessive amount of nucleotide fixations during domestication. Artificial selection during soybean domestication has modified a number of traits including seed size, seed color, plant height and prostrate habitat, shaping the domestication syndrome [11,43]. To analyze the effects of nucleotide fixation during artificial selection, we focused on genes within QTLs responsible for domestication-related traits (www.soybase.org), such as oil content, pod number, lodging, plant height, *etc*. Meta-analysis of these QTLs revealed that 51 of them responsible for 13 traits and 33 for 11 traits were affected by nucleotide fixation in early domestication and modern improvement, respectively (Additional file 1: Table S2, S3). Total QTL regions were narrowed down from 214.9 Mb to 8.1 Mb assisted by selective signals. Analysis of related genes, as well as their orthologs through comparative genomics, could provide information on their potential functions under artificial selection.

As an agriculturally important trait, grain filling makes a significant contribution to grain weight [44]. The gene *Grain Incomplete Filling 1* (*GIF1*) was detected to be responsible and associated with this domestication syndrome [45]. It was reported to encode a cell-wall invertase required for carbon partitioning during early grain filling in rice. A selective gene *Glyma03g35520* with nucleotide fixation in domestication is an ortholog of *GIF1* and this gene was involved in the carbohydrate metabolism pathway by searching KEGG (Additional file 1: Table S4). Besides, this gene was covered by the QTLs responsible for lodging and pod number. It indicates that *Glyma03g35520* is a potential candidate gene, which could be used for further soybean breeding.

Flower and pod numbers per plant are important agronomic traits for grain yield in soybean. To detect the genes involved in flower and pod numbers will help to understand the genetic basis of soybean yield [46]. Two genes, *Glyma07g05470* and *Glyma07g05480*, with nucleotide fixation introduced in improvement, are orthologs of *COMT2* gene encoding caffeic acid 3-O-methyltransferase (Additional file 1: Table S5). It differentially expressed in hair cells of growing pod, the possible location of vanillin biosynthesis [47]. Another five selective genes with nucleotide fixation mediated by domestication and improvement encode a kind of protein responsible for the transportation of inositol. These genes were covered by QTLs responsible for seed-coat color, protein and pod number. Previous study showed that the total number of mature pods considerably higher due to the application of inositol, indicating the positive effect in pod number [48]. It suggested that deficiency of lignin biosynthesis resulted in growth reduction and dwarfing [49]. The gene *Glyma13g21010* is linked to marker Sat103 that associate with seed weight. As an orthologs of *NifU* gene, it is required for full activation of nitrogenase catalytic components [50]. *NifU* protein has been suggested to either mobilize the Fe necessary for nitrogenase Fe-S cluster formation or provide an intermediate Fe-S cluster assembly site [51]. In addition, the gene was reported to be related to seed weight [52]. As nitrogen fixation is imperative in soybean growth, *Glyma13g21010* gene could also be a putative candidate gene responsible for seed weight through activating biological nitrogen fixation.

The flowering of soybean represents the transition from a vegetative state to a reproductive state, making a contribution to the yield. Meta-analysis of QTLs identified 14 selective genes with non-synonymous nucleotide fixation responsible for flower number in soybean. Carbon fixation in the process of photosynthesis is pivotal to soybean production. Seven selective genes with nucleotide fixation were involved in photosynthesis or photosystem. Besides, two selective genes *Glyma03g36970* and *Glyma19g39620* with nucleotide fixation were identified as orthologs of *Luminidependens*, which is involved in the timing of flowering in Arabidopsis [53].

Interestingly, 63 and 27 selective genes with nucleotide fixation in domestication and improvement, respectively, were annotated to be, or related with transcription factors. Analysis of all the genes subject to artificial selection with agriGO [54] also told an accumulation of transcription factors by Fisher’s exact test and the permutation test (Additional file 1: Table S6). Most of the genes cloned to date that responsible for domestication related traits in crops were proved to be transcription factors, such as *teosinte branched 1* (*tb1*) [55], shattering (*sh4*) [56], six-rowed spike (*vsr1*) [57,58], *etc*. It is probably because the human mediated domestication history was momentary compared with the long natural evolution; changing the transcription factors probably the easiest way happened to affect the agricultural traits of interest. However, putative candidate genes underlying these domestication-targeted phenotypes have diverse functions, which need to be validated by further experiments.

### Plant-pathogen interaction affected by artificial selection

**Figure 5:**
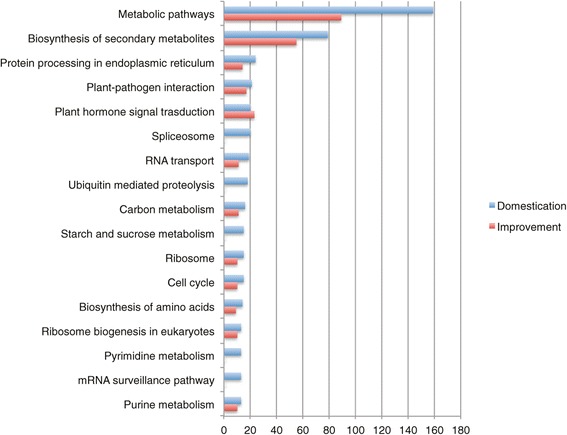
**Functional annotation of selective genes with nucleotide fixation introduced in early domestication and modern improvement.**

Domestication caused complex morphological and physiological changes in soybeans. Annotated by the KEGG and agriGO database, selective genes were associated with different biological functions, among which, plant-pathogen interaction, sequence-specific DNA binding, phenylpropanoid biosynthesis, starch and sucrose metabolism are over-represented categories (Figure 5; Additional file 6: Figure S5). Plant-pathogen interactions are conducted between a pathogen and the host plant. In nature, plants are generally resistant to most invading pathogens due to innate ability to recognize them through successful defenses. When an exception happens, a pathogen would cause diseases in its host [59]. However, pathogens could also cause diseases if they have evolved to evade detection or suppress host defense mechanisms, or both. The effects of plant-pathogen interactions are of particular relevance during early domestication events on agricultural systems [60]. Thus, understanding the genetic basis of why a certain pathogen causes disease in its host plant instead of others has long intrigued and motivated plant pathologists.

A total of 37 selective genes with nucleotide fixation were involved in plant-pathogen interactions (Additional file 7: Figure S6). Of them, two selective genes *Glyma14g36511* and *Glyma08g12560* with nucleotide fixation are orthologs of *RPS2* gene. The disease resistance gene *RPS2* was isolated using positional cloning and further screen for susceptible mutant [61,62]. The *RPS2* protein contains two characteristics of a large family of plant *R* genes: a nucleotide-binding site and a leucine-rich repeat region [63]. It is consistent with previous report that *RPS2* locus exhibit selection signals by examining a worldwide sample of 27 Arabidopsis accessions, and the N-terminal part of the leucine-rich repeat region was a probable target of selection [64].

Cyclic nucleotide-gated ion (CNG) channels are ion channels that function in the pathogen signaling cascade by facilitating Ca^2+^ uptake into the cytosol [65]. Two selective genes with nucleotide fixation were detected to encode CNG channels. The topology of their proteins was predicted using TMHMM, which is based on a hidden Markov model [66]. The two genes encode transmembrane proteins with nucleotide fixation located outside the membrane (Additional file 8: Figure S7). Besides, eight selective genes are orthologs of transmembrane receptor kinase *FLS2*, which acts as pathogen-associated molecular pattern signals in triggering the innate immune response [67].

In addition, the category of terpene synthase activity was also enriched with six selective genes involved in (Additional file 1: Table S6). Terpenes are one of the most important defensive plant compounds against herbivores and pathogens [68]. Recently, a new monoterpene synthase gene *GmNES* was identified and characterized in soybean [69]. Its transcription was up-regulated in soybean when infested with cotton leafworm. Our analysis indicates the gene was possibly affected by artificial selection during soybean domestication.

## Discussion

### Nucleotide fixation was crucial in soybean divergence

Domestication led to significant morphological divergence between cultivated and wild soybeans. Wild soybean exhibits, for example, twining and vine stem, severer shattering, impermeable seed coats, pod cracking sensitivity, small seeds, and low oleic acid, all of which are seldom observed in cultivars [70]. Deciphering how cultivated soybean have been transformed from its wild ancestor is advantageous both from genetic and evolutionary perspectives. With the available sequencing data, we comprehensively estimated the saturation number of SNVs in soybean germplasm and detected a set of candidate genes showing artificial selection signals. To some degree, analysis of artificial selection and nucleotide fixation unravels the mystery of soybean domestication and subsequent improvement. Based on nucleotide fixation, our analysis supports a single evolutionary origin of domesticated soybean. During domestication, only lines with certain agriculturally important traits were selected, resulting in a genome-wide reduction of genetic diversity or so-called selective sweep in cultivated crops [42,71,72]. One possible explanation for the reduction is that an excess of nucleotide fixation happened in cultivars compared to wild soybeans.

Meta-analysis of QTLs responsible for domestication related traits and the selective genes provided insights into the role of nucleotide fixation played in morphological differentiation between wild and cultivated soybeans. Using comparative genomics, an amount of genes was found to be orthologs of those whose function was validated and responsible for corresponding traits in other plants. Nucleotide fixation happened in those genes responsible for agronomically important traits. Although traditional linkage and association mapping were used to dissect these traits, they failed to detect genetic changes caused by domestication and improvement [73]. Our analysis here provides valuable information for further QTL mapping and will facilitate molecular assisted selection in soybean breeding practice.

### Artificial selection accelerates nucleotide fixation

Domestication was an evolutionary process where the characters of interests were selected, such as loss of seed dispersal, higher yield and increasing abiotic resistance. The detection of selective loci during crop domestication contributes to modern breeding efforts and the opportunity to improve genomic selection models [74]. Recently, genome-wide scans based on genetic bottlenecks have been successfully applied to detect footprints of selection in plants by surveying both natural and cultivated species [19,75,76]. Artificial selection of a beneficial mutation will lead to an elevated frequency in a population. Eventually, allele frequencies will be skewed and nucleotide fixation happened after plant domestication. Our analysis focused on to what degree nucleotide fixation was caused by artificial selection during soybean domestication.

More nucleotide fixation happened in cultivars than those in wild soybeans, indicating that artificial selection was much stronger than natural selection. However, the effective population size of cultivated soybeans was substantially reduced during domestication [77], which could make a nucleotide seem to be fixed in cultivars. That mainly explains why nucleotide fixations were observed in cultivars across the soybean genome. Considering nucleotide fixation accumulated in footprints of domestication and improvement, artificial selection probably accelerated the occurrence of fixation in soybean breeding activities. Even thought, some of them could be also caused by the shrinking population size, especially when different haplotypes shaped in those selective sweeps. These fixations are extremely hard to be distinguished in current samples.

Morphological transition can be achieved by a mutation at a single locus [78,79], and artificial selection can rapidly change domestication targeted phenotypes within 20 generations [31,80]. Domestication could be a rapid instead of a slow or gradual process, given strong selective pressures and a suitable genetic architecture. This was supported by the severe reduction of genetic diversity and large selective sweeps. In the process of domestication, any mutations detrimental to the traits of interests were eliminated immediately, whereas those advantageous ones were strongly selected, diffused and eventually fixed in a population. The environments wild soybeans grow in are various and usually harsh, resulting in diversifying selection instead of strong directional selection. What’s more, selection intensity imposed by natural selection was disparate in diverse habitats. These reasons also explain why artificial selection was much stronger than natural selection in crop domestication.

### Evolutionary perspective of nucleotide fixation

A long-term goal of crop genomics is to determine to what extent artificial selection impacts genomic variation patterns within and between populations. There are both genetic and statistical approaches to detect signals of hitchhiking caused selective sweeps [13]. The hitchhiking effect is contingent on the nature of genetic variations and how selection acts on them. Generally, there are at least three evolutionary routes by which a novel mutation may fix: drift to fixation for nearly neutral mutation; rapidly sweep to fixation, so-called hard sweep for beneficial mutation; and soft sweep to fixation for those initially neutral but later become beneficial for some reason. Affected by artificial selection, a pre-exist mutation which became beneficial during domestication rapidly increased in frequency toward nucleotide fixation, as what we found in our analysis. When traits of interests during domestication were determined by multiple adaptive mutations at the same locus, artificial selection usually generates soft rather hard selective sweeps. Many studies focus on hard sweeps in which only a single adaptive haplotype was skewed to fixation in the population [81], whereas multiple adaptive haplotypes formed simultaneously in a soft sweep. Lots of nucleotide fixations happened within quantitative traits, indicating the corresponding traits of interests were incrementally changed at various causal loci. As a consequence, these sweeps related with artificial selection are likely to be both soft and incomplete. In soybean, some traits related to yield were selected, such as seed weight, seed blooming and prostrate habit, for which these are usually major QTLs responsible. Nevertheless, during intensive breeding human pursuits quality related traits such as protein content and lipid content, for which there are lots of small effect QTLs responsible. Analysis of nucleotide fixation indicates that more soft selective sweeps happened in extensive breeding than in early domestication in soybean, which still needs further investigation.

## Conclusion

We integrated the available sequencing accessions to describe a whole picture of soybean genetic diversity, artificial selection and concomitant nucleotide fixation. There are approximately 9.8 million SNVs in soybean germplasm, of which about 5.3 million reserved in cultivars. The genetic diversity was reduced by 37.5% in early domestication and subsequently reduced by 8.3% in genetic improvement. A total of 2,255 and 1,051 genes were involved in early domestication and subsequent improvement, respectively. Both processes introduced about 0.1 million nucleotide fixations, which contributed to the divergence of wild and cultivated soybeans. Artificial selection probably accelerated the occurrence of nucleotide fixation, which affected some agronomic traits, as well as related biological pathways such as plant-pathogen interaction.

## Methods

### Data collection and SNP detection

The sequenced soybean accessions representing 31 wild, 15 landrace, and 24 elites were described in several published papers [7-10]. These accessions originate from large ecological regions in China and South Korea. All sequence reads were downloaded in Sequence Read Archive (SRA) under accession number SRP015830, SRA020131 SRA009252, and ERP002622. These reads were then mapped to the soybean reference (*Glycine max* var. Williams 82, Phytozome v9.0) with SOAP2 software [15]. PCR duplication in each sequencing library was removed before SNV calling.

In the SNV calling process, genotype likelihood of each genomic locus was first calculated with Bayesian theory implemented in SOAPsnp [16]. The genotype with the highest probability at each site was selected with a quality value to create a consensus sequence for each individual. High quality SNVs were obtained with certain criteria such as sequencing depth, copy number (<=1.5), quality value (>20) and the rank sum test.

### Detection of artificial selection signals

As described in previous report [10], we used two outlier approaches to detect signals of artificial selection. Using a 20 kb sliding window with a 2 kb step-size, we calculated *θ*_*π*_ and Tajima’s *D* between wild and cultivated groups. Those regions showing significantly low *θ*_*π.cultivated*_/*θ*_*π.wild*_ and low *D* values (*Z* test, *P* < 0.05 for both) in cultivars were treated as putative selection signals. Besides, we chose the population branch statistic [27] on the basis of *F*_*st*_ to infer the selective footprints from landrace to elite cultivar, considering the very short divergence time between them.

### Identification of nucleotide fixation

We screened the SNVs located in the regions showing signals of artificial selection. Short reads of each individual were re-aligned to the reference for individual genotyping at each SNV. The likelihood of individual genotypes was calculated and then the allele type with the maximum likelihood was allocated back to each individual. If a SNV has a unique genotype in all wild soybeans or in cultivars, it will be identified as a nucleotide fixation locus.

### PCA and phylogenetic analysis

Using the principal component analysis (PCA), the population subdivision pattern was then inferred [82]. We constructed a phylogenetic tree by a neighbor joining method in the software PHYLIP (version 3.68) [83]. A total of 1,000 replicates generated the bootstrap values.

### Enrichment of selective genes

The functions of selective genes were analyzed with KEGG (www.genome.jp/kegg/) and agriGO (http://bioinfo.cau.edu.cn/agriGO/), and the results were displayed using a Cytoscape plugin BiNGO [84]. For enrichment *P* value (<0.05) was calculated using Fisher’s exact test and Permutation test. For multiple hypotheses testing, false discovery rate correction of Benjamini and Hochberg method was used to reduce false negatives.

### Inferring protein topology

We predicted transmembrane protein topology with a hidden Markov model (TMHMM) to infer the protein topology with default parameters [66] (http://www.cbs.dtu.dk/services/TMHMM/).

## Additional files

Additional file 1: Table S1. Summary of sequencing soybean accessions collected from publications. **Table S2**. Meta-analysis of the published QTLs responsible for agriculturally important traits and selective genes with nucleotide fixation during early domestication. **Table S3**. Meta-analysis of the published QTLs responsible for agriculturally important traits and selective genes with nucleotide fixation during modern improvement. **Table S4**. Functional analysis of the selective genes with nucleotide fixation during early domestication. **Table S5**. Functional analysis of the selective genes with nucleotide fixation during modern improvement. **Table S6**. Functional analysis of the selective genes with nucleotide fixation based on agriGO. Additional file 2: Figure S1. Fixed SNP distribution on each chromosome. Additional file 3: Figure S2. The allele frequency of SNVs in wild soybeans that were fixed in cultivars. The allele frequency < 0.1 was underestimated in SNV calling to improve accuracy. Additional file 4: Figure S3. (A) PCA and (B) phylogenetic tree among soybean accessions based on nucleotide fixation. Additional file 5: Figure S4. The accumulated KEGG pathway in the genes with nucleotide fixation in wild soybeans. Additional file 6: Figure S5. Over-represented GO categories in the selective genes with nucleotide fixation (Fisher’s exact test < 0.05 and false discovery rate (FDR) < 0.05). Additional file 7: Figure S6. Selective genes with nucleotide fixation involved in plant hormone signal transduction pathway. Red: affected by early domestication; Green: affected both by domestication and improvement. Additional file 8: Figure S7. The protein topology CNG channels involved in plant-pathogen interaction pathway. The stars denote nucleotide fixation in the protein.

## Abbreviations

QTL: Quantitative Trait Loci
SNV: Single nucleotide variation
PCA: Principle component analysis
KEGG: Kyoto Encyclopedia of Genes and Genomes
*GIF1*: *Grain Incomplete Filling 1*
CNG: Cyclic Nucleotide-Gated ion
*tb1*: *teosinte branched 1*

## References

[1] Hymowiltz T, Boerma HR, Specht JE (2004). Speciation and cytogenetics. Soybeans: Improvement, production, and uses.

[2] Kato S, Sayama T, Fujii K, Yumoto S, Kono Y, Hwang T-Y (2014). A major and stable QTL associated with seed weight in soybean across multiple environments and genetic backgrounds. Theor Appl Genet.

[3] Saitoh K, Nishimura K, Kuroda T (2008). Comparisons of growth and photosynthetic characteristics between wild and cultivated types of soybeans.

[4] Liu B, Fujita T, Yan ZH, Sakamoto S, Xu D, Abe J (2007). QTL Mapping of Domestication-related Traits in Soybean (*Glycine max*). Ann Bot.

[5] Vaughan DA, Balazs E, Heslop-Harrison JS (2007). From crop domestication to super-domestication. Ann Bot.

[6] Schmutz J, Cannon SB, Schlueter J, Ma J, Mitros T, Nelson W (2010). Genome sequence of the palaeopolyploid soybean. Nature.

[7] Lam H-M, Xu X, Liu X, Chen W, Yang G, Wong F-L (2010). Resequencing of 31 wild and cultivated soybean genomes identifies patterns of genetic diversity and selection. Nat Genet.

[8] Chung WH, Jeong N, Kim J, Lee WK, Lee YG, Lee SH (2014). Population structure and domestication revealed by high-depth resequencing of Korean cultivated and wild soybean genomes. DNA Res.

[9] Kim MY, Lee S, Van K, Kim T-H, Jeong S-C, Choi I-Y (2010). Whole-genome sequencing and intensive analysis of the undomesticated soybean (*Glycine soja* Sieb. and Zucc.) genome. Proc Natl Acad Sci U S A.

[10] Li Y-H, Zhao S-C, Ma J-X, Li D, Yan L, Li J (2013). Molecular footprints of domestication and improvement in soybean revealed by whole genome re-sequencing. BMC Genomics.

[11] Doebley JF, Gaut BS, Smith BD (2006). The molecular genetics of crop domestication. Cell.

[12] Qi X, Li M-W, Xie M, Liu X, Ni M, Shao G (2014). Identification of a novel salt tolerance gene in wild soybean by whole-genome sequencing. Nat Commun.

[13] Nielsen R (2005). Molecular signatures of natural selection. Annu Rev Genet.

[14] Innan H, Kim Y (2004). Pattern of polymorphism after strong artificial selection in a domestication event. Proc Natl Acad Sci U S A.

[15] Li R, Yu C, Li Y, Lam T-W, Yiu S-M, Kristiansen K (2009). SOAP2: an improved ultrafast tool for short read alignment. Bioinformatics.

[16] Li R, Li Y, Fang X, Yang H, Wang J, Kristiansen K (2009). SNP detection for massively parallel whole-genome resequencing. Genome Res.

[17] Wang J, Wang W, Li R, Li Y, Tian G, Goodman L (2008). The diploid genome sequence of an Asian individual. Nature.

[18] Xia QQ, Guo YY, Zhang ZZ, Li DD, Xuan ZZ, Li ZZ (2009). Complete resequencing of 40 genomes reveals domestication events and genes in silkworm (*Bombyx*). Science.

[19] Xu X, Liu X, Ge S, Jensen JD, Hu F, Li X (2012). Resequencing 50 accessions of cultivated and wild rice yields markers for identifying agronomically important genes. Nat Biotech.

[20] Hyten DL, Song Q, Zhu Y, Choi I-Y, Nelson RL, Costa JM (2006). Impacts of genetic bottlenecks on soybean genome diversity. Proc Natl Acad Sci U S A.

[21] Tajima F (1983). Evolutionary relationship of DNA sequences in finite populations. Genetics.

[22] Watterson GA (1975). On the number of segregating sites in genetical models without recombination. Theor Popul Biol.

[23] Tanksley SD (1997). Seed banks and molecular maps: unlocking genetic potential from the wild. Science.

[24] Nielsen R, Williamson S, Kim Y, Hubisz MJ, Clark AG, Bustamante C (2005). Genomic scans for selective sweeps using SNP data. Genome Res.

[25] Tian F, Stevens NM, Buckler ES (2009). Tracking footprints of maize domestication and evidence for a massive selective sweep on chromosome 10. Proc Natl Acad Sci U S A.

[26] Huang X, Kurata N, Wei X, Wang Z-X, Wang A, Zhao Q (2012). A map of rice genome variation reveals the origin of cultivated rice. Nature.

[27] Yi X, Liang Y, Huerta-Sanchez E, Jin X, Cuo ZXP, Pool JE (2010). Sequencing of 50 human exomes reveals adaptation to high altitude. Science.

[28] Tajima F (1989). Statistical method for testing the neutral mutation hypothesis by DNA polymorphism. Genetics.

[29] Weir BS, Cockerham CC. Estimating F-statistics for the analysis of population structure. Evolution. 1984;1358–1370. 10.1111/j.1558-5646.1984.tb05657.x 28563791

[30] Bamshad M, Wooding SP (2003). Signatures of natural selection in the human genome. Nat Rev Genet.

[31] Purugganan MD, Fuller DQ (2009). The nature of selection during plant domestication. Nature.

[32] Yang Z, Nielsen R (2000). Estimating synonymous and nonsynonymous substitution rates under realistic evolutionary models. Mol Biol Evol.

[33] Rosenberg NA, Pritchard JK, Weber JL, Cann HM, Kidd KK, Zhivotovsky LA (2002). Genetic structure of human populations. Science.

[34] McNally KL, Childs KL, Bohnert R, Davidson RM, Zhao K, Ulat VJ (2009). Genomewide SNP variation reveals relationships among landraces and modern varieties of rice. Proc Natl Acad Sci U S A.

[35] Molina J, Sikora M, Garud N, Flowers JM, Rubinstein S, Reynolds A (2011). Molecular evidence for a single evolutionary origin of domesticated rice. Proc Natl Acad Sci U S A.

[36] Xu D, Abe J, Gai J, Shimamoto Y (2002). Diversity of chloroplast DNA SSRs in wild and cultivated soybeans: evidence for multiple origins of cultivated soybean. Theor Appl Genet.

[37] Guo J, Wang Y, Song C, Zhou J, Qiu L, Huang H (2010). A single origin and moderate bottleneck during domestication of soybean (*Glycine max*): implications from microsatellites and nucleotide sequences. Ann Bot.

[38] Tajima F (1990). Relationship between DNA polymorphism and fixation time. Genetics.

[39] Fuller DQ (2007). Contrasting patterns in crop domestication and domestication rates: recent archaeobotanical insights from the Old World. Ann Bot.

[40] Grant MR, Godiard L, Straube E, Ashfield T, Lewald J, Sattler A (1995). Structure of the Arabidopsis RPM1 gene enabling dual specificity disease resistance. Science.

[41] Ashfield T, Redditt T, Russell A, Kessens R, Rodibaugh N, Galloway L (2014). Evolutionary relationship of disease resistance genes in soybean and Arabidopsis specific for the *pseudomonas syringae* effectors AvrB and AvrRpm1. Plant Physiol.

[42] Diamond J (2002). Evolution, consequences and future of plant and animal domestication. Nature.

[43] Gepts P (2002). A comparison between crop domestication, classical plant breeding, and genetic engineering. Crop Sci.

[44] Takai T (2005). Time-related mapping of quantitative trait loci controlling grain-filling in rice (*Oryza sativa* L.). J Exp Bot.

[45] Wang E, Wang J, Zhu X, Hao W, Wang L, Li Q (2008). Control of rice grain-filling and yield by a gene with a potential signature of domestication. Nat Genet.

[46] Zhang D, Cheng H, Wang H, Zhang H, Liu C, Yu D (2010). Identification of genomic regions determining flower and pod numbers development in soybean (*Glycine max* L). J Genet Genomics.

[47] Grimmig B, Matern U (1997). Structure of the parsley caffeoyl-CoA O-methyltransferase gene, harbouring a novel elicitor responsive *cis*-acting element. Plant Mol Biol.

[48] Yang Z, Xin D, Liu C, Jiang H, Han X, Sun Y (2013). Identification of QTLs for seed and pod traits in soybean and analysis for additive effects and epistatic effects of QTLs among multiple environments. Mol Genet Genomics.

[49] Li Y-H, Li W, Zhang C, Yang L, Chang R-Z, Gaut BS (2010). Genetic diversity in domesticated soybean (*Glycine max*) and its wild progenitor (*Glycine soja*) for simple sequence repeat and single-nucleotide polymorphism loci. New Phytologist.

[50] Hwang DM, Dempsey A, Tan KT, Liew CC (1996). A modular domain of NifU, a nitrogen fixation cluster protein, is highly conserved in evolution. J Mol Evol.

[51] Yuvaniyama P, Agar JN, Cash VL, Johnson MK, Dean DR (2000). NifS-directed assembly of a transient [2Fe-2S] cluster within the NifU protein. Proc Natl Acad Sci U S A.

[52] Atta S, Maltese S, Cousin R (2004). Protein content and dry weight of seeds from various pea genotypes. Agronomie.

[53] Lee I, Aukerman MJ, Gore SL, Lohman KN, Michaels SD, Weaver LM (1994). Isolation of *LUMINIDEPENDENS*: a gene involved in the control of flowering time in Arabidopsis. Plant Cell.

[54] Du Z, Zhou X, Ling Y, Zhang Z, Su Z (2010). agriGO: a GO analysis toolkit for the agricultural community. Nucleic Acids Res.

[55] Doebley J, Stec A, Hubbard L (1997). The evolution of apical dominance in maize. Nature.

[56] Li C (2006). Rice domestication by reducing shattering. Science.

[57] Komatsuda T, Pourkheirandish M, He C, Azhaguvel P, Kanamori H, Perovic D (2007). Six-rowed barley originated from a mutation in a homeodomain-leucine zipper I-class homeobox gene. Proc Natl Acad Sci U S A.

[58] Ramsay L, Comadran J, Druka A, Marshall DF, Thomas WTB, Macaulay M (2011). *INTERMEDIUM-C*, a modifier of lateral spikelet fertility in barley, is an ortholog of the maize domestication gene *TEOSINTE BRANCHED 1*. Nat Genet.

[59] Staskawicz BJ (2000). Genetics of plant-pathogen interactions specifying plant disease resistance. Plant Physiol.

[60] Dodds PN, Rathjen JP (2010). Plant immunity: towards an integrated view of plant-pathogen interactions. Nat Rev Genet.

[61] Bent AF, Kunkel BN, Dahlbeck D, Brown KL, Schmidt R, Giraudat J (1994). RPS2 of *Arabidopsis thaliana*: a leucine-rich repeat class of plant disease resistance genes. Science.

[62] Kunkel BN, Bent AF, Dahlbeck D, Innes RW, Staskawicz BJ (1993). RPS2, an Arabidopsis disease resistance locus specifying recognition of Pseudomonas syringae strains expressing the avirulence gene avrRpt2. Plant Cell.

[63] Luck JE, Lawrence GJ, Dodds PN, Shepherd KW, Ellis JG (2000). Regions outside of the leucine-rich repeats of flax rust resistance proteins play a role in specificity determination. Plant Cell.

[64] Mauricio R, Stahl EA, Korves T, Tian D, Kreitman M, Bergelson J (2003). Natural selection for polymorphism in the disease resistance gene Rps2 of *Arabidopsis thaliana*. Genetics.

[65] Ma W (2011). Roles of Ca2+ and cyclic nucleotide gated channel in plant innate immunity. Plant Sci.

[66] Krogh A, Larsson B, von Heijne G, Sonnhammer EL (2001). Predicting transmembrane protein topology with a hidden Markov model: application to complete genomes. J Mol Biol.

[67] Chinchilla D, Bauer Z, Regenass M, Boller T, Felix G (2006). The Arabidopsis receptor kinase FLS2 binds flg22 and determines the specificity of flagellin perception. Plant Cell.

[68] Wittstock U, Gershenzon J (2002). Constitutive plant toxins and their role in defense against herbivores and pathogens. Curr Opin Plant Biol.

[69] Zhang M, Liu J, Li K, Yu D (2013). Identification and characterization of a novel monoterpene synthase from soybean restricted to neryl diphosphate precursor. PLoS One.

[70] Chen Y, Nelson RL (2004). Identification and characterization of a white-flowered wild soybean plant. Crop Sci.

[71] Buckler ES, Thornsberry JM, Kresovich S (2001). Molecular diversity, structure and domestication of grasses. Genet Res.

[72] Burger JC, Chapman MA, Burke JM (2008). Molecular insights into the evolution of crop plants. Am J Bot.

[73] Varshney RK, Hoisington DA, Tyagi AK (2006). Advances in cereal genomics and applications in crop breeding. Trends Biotechnol.

[74] Morrell PL, Buckler ES, Ross-Ibarra J (2011). Crop genomics: advances and applications. Nat Rev Genet.

[75] Hufford MB, Xu X, van Heerwaarden J, Pyhäjärvi T, Chia J-M, Cartwright RA (2012). Comparative population genomics of maize domestication and improvement. Nat Genet.

[76] Morris GP, Ramu P, Deshpande SP, Hash CT, Shah T, Upadhyaya HD (2013). Population genomic and genome-wide association studies of agroclimatic traits in sorghum. Proc Natl Acad Sci U S A.

[77] Tang H, Sezen U, Paterson AH (2010). Domestication and plant genomes. Curr Opin Plant Biol.

[78] Doebley J, Stec A (1993). Inheritance of the morphological differences between maize and teosinte: comparison of results for two F2 populations. Genetics.

[79] Doebley J (2004). The genetics of maize evolution. Annu Rev Genet.

[80] HILLMAN GC, DAVIES MS (1990). Domestication rates in wild-type wheats and barley under primitive cultivation. Biol J Linn Soc.

[81] Sabeti PC, Varilly P, Fry B, Lohmueller J, Hostetter E, Cotsapas C (2007). Genome-wide detection and characterization of positive selection in human populations. Nature.

[82] Patterson N, Price AL, Reich D (2006). Population structure and eigenanalysis. PLoS Genet.

[83] Felsenstein J (1989). PHYLIP - Phylogeny inference package (version 3.2). Cladistics.

[84] Maere S, Heymans K, Kuiper M (2005). BiNGO: a Cytoscape plugin to assess overrepresentation of Gene Ontology categories in Biological Networks. Bioinformatics.

