# Supplementary figures and images for: Impacts of nucleotide fixation during soybean domestication and improvement
Source: BMC Plant Biol. 2015 Mar 8;15:81. doi: 10.1186/s12870-015-0463-z (PMC4358728; doi:10.1186/s12870-015-0463-z)

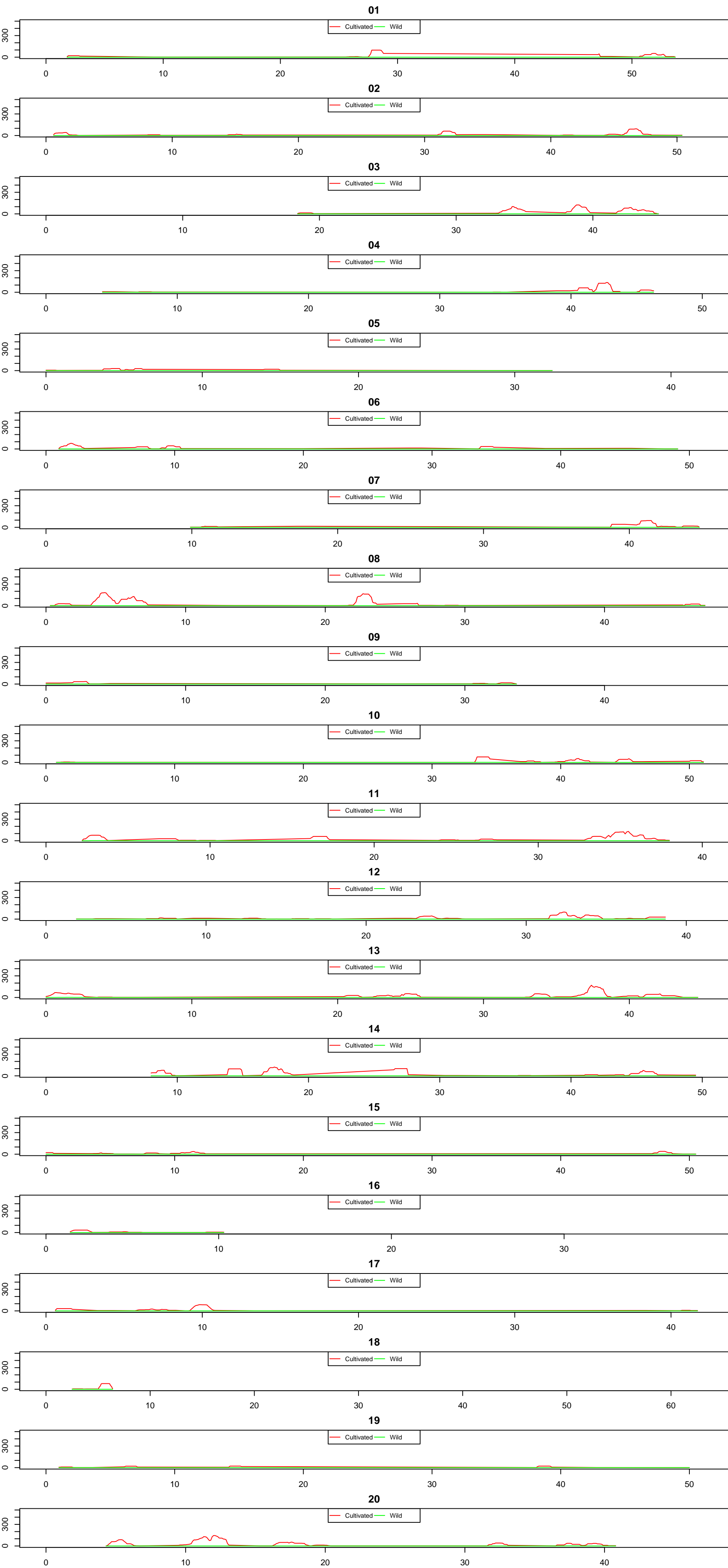

Supplement: Additional file 2: Figure S1. — Fixed SNP distribution on each chromosome. [file 12870_2015_463_MOESM2_ESM.pdf]

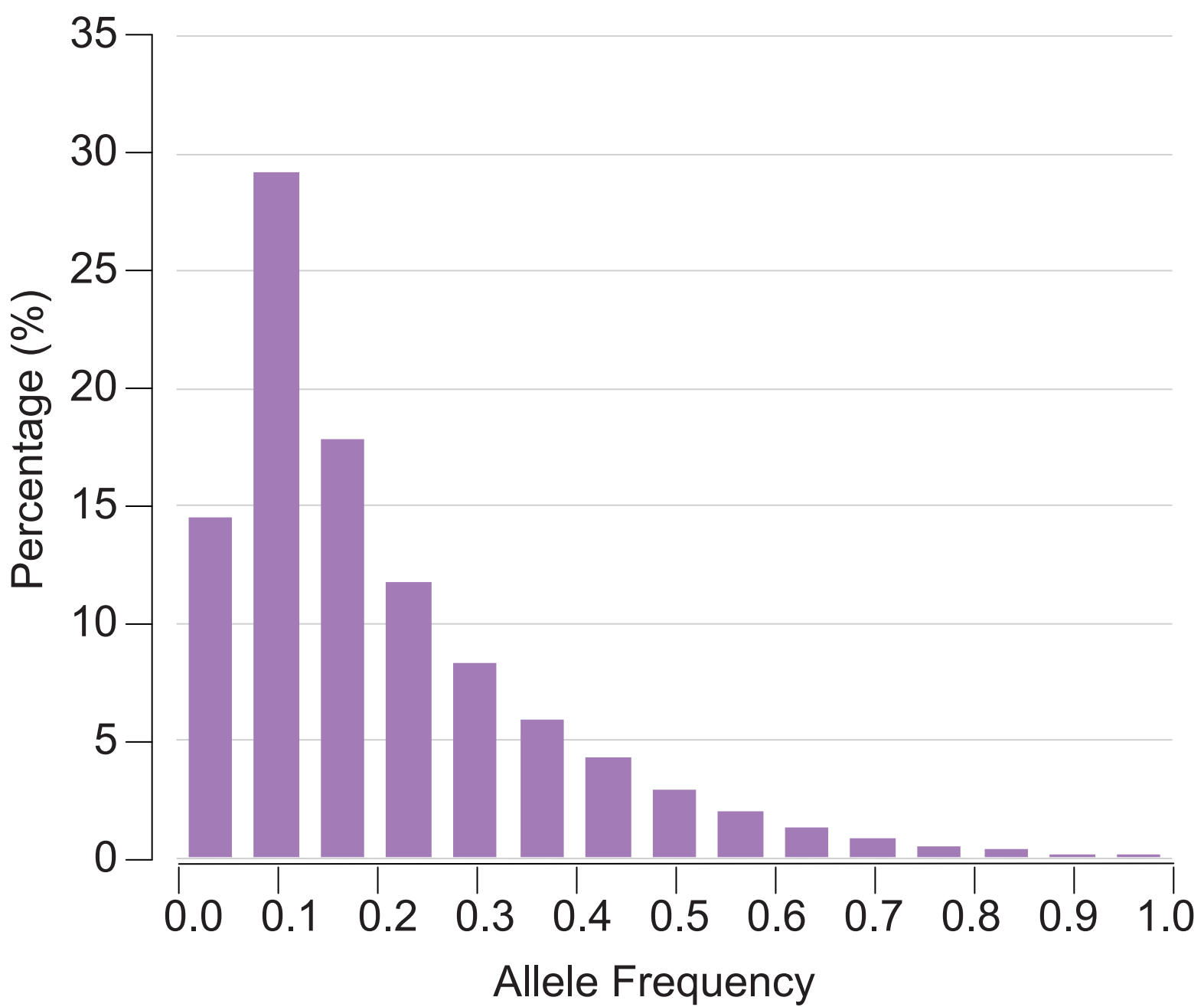

Supplement: Additional file 3: Figure S2. — The allele frequency of SNVs in wild soybeans that were fixed in cultivars. The allele frequency < 0.1 was underestimated in SNV calling to improve accuracy. [file 12870_2015_463_MOESM3_ESM.pdf]

A.

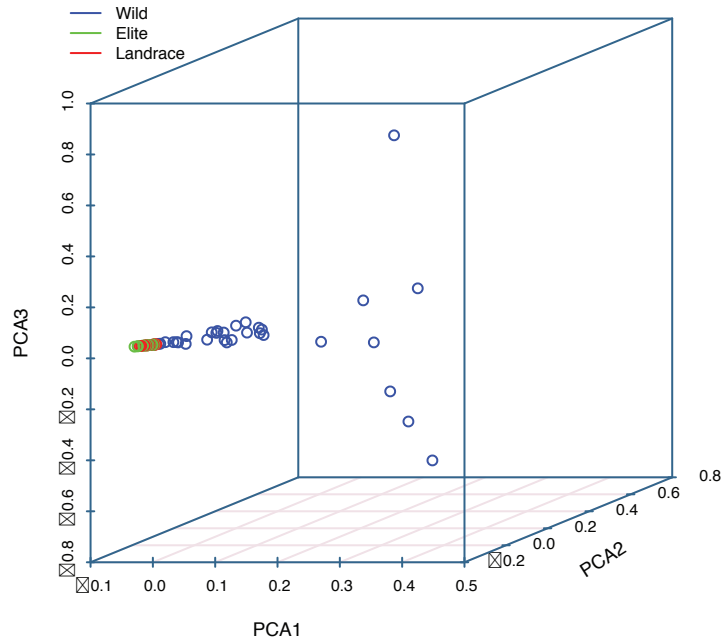

B.

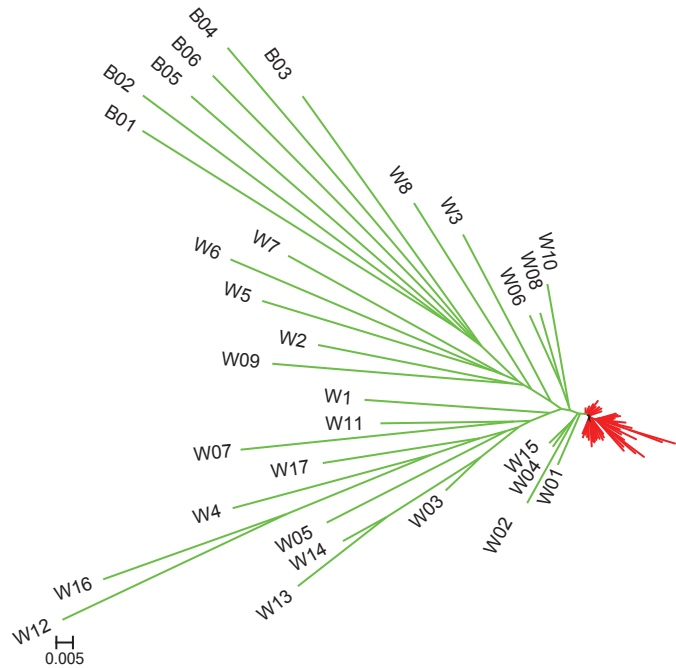

Supplement: Additional file 4: Figure S3. — (A) PCA and (B) phylogenetic tree among soybean accessions based on nucleotide fixation. [file 12870_2015_463_MOESM4_ESM.pdf]

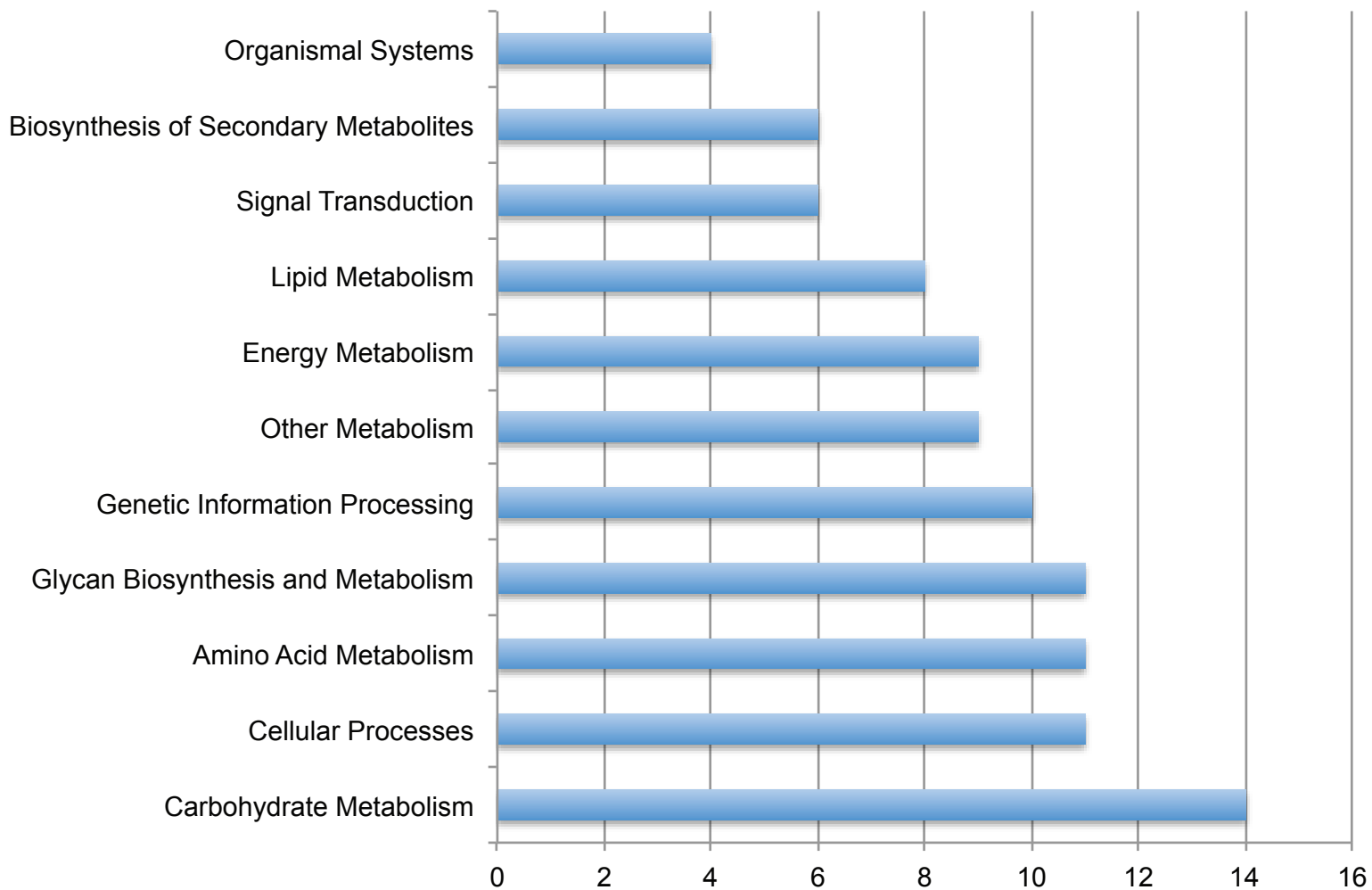

Supplement: Additional file 5: Figure S4. — The accumulated KEGG pathway in the genes with nucleotide fixation in wild soybeans. [file 12870_2015_463_MOESM5_ESM.pdf]

A.

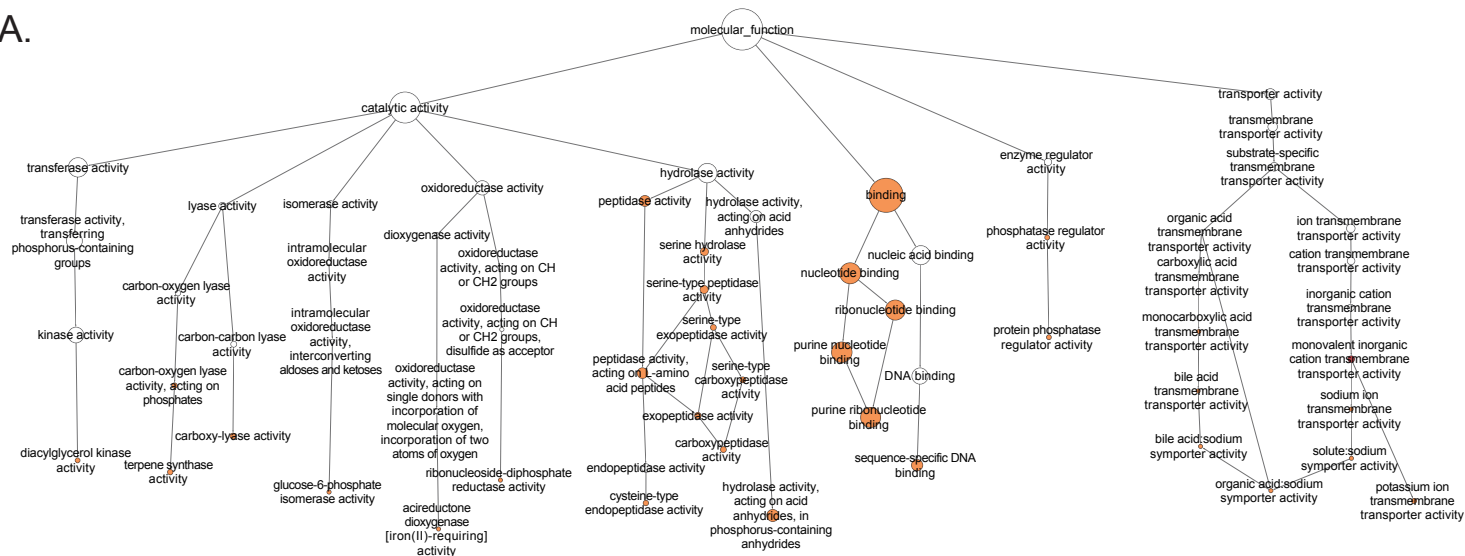

B.

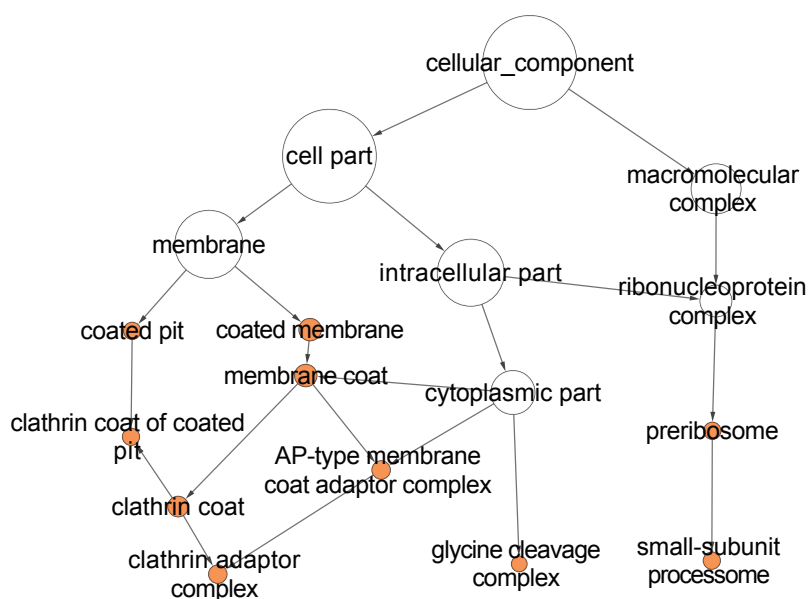

C.

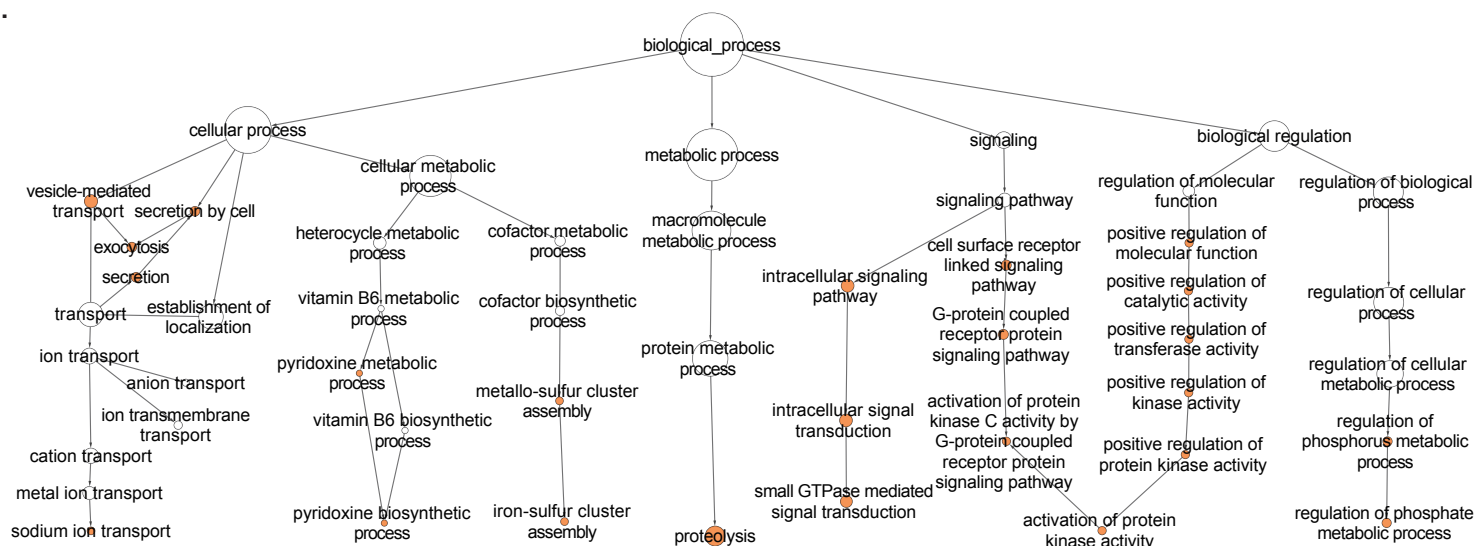

Supplement: Additional file 6: Figure S5. — Over-represented GO categories in the selective genes with nucleotide fixation (Fisher’s exact test < 0.05 and false discovery rate (FDR) < 0.05). [file 12870_2015_463_MOESM6_ESM.pdf]

PLANT-PATHOGEN INTERACTION

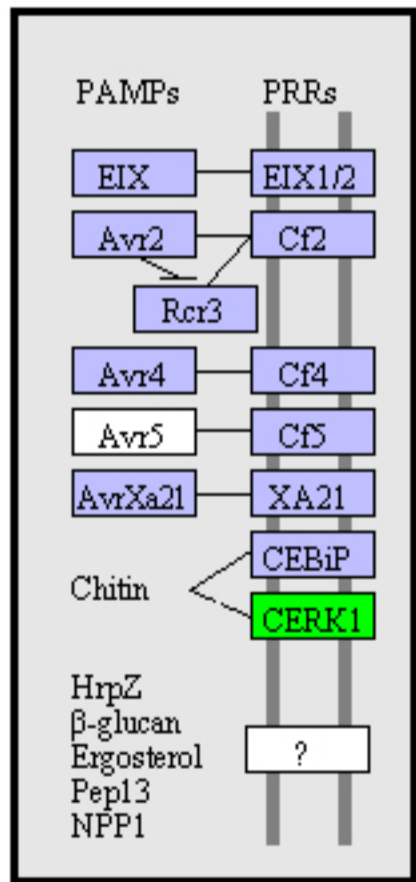

Supplement: Additional file 7: Figure S6. — Selective genes with nucleotide fixation involved in plant hormone signal transduction pathway. Red: affected by early domestication; Green: affected both by domestication and improvement. [file 12870_2015_463_MOESM7_ESM.pdf]

TMHMM posterior probabilities for Glyma12g29190.1

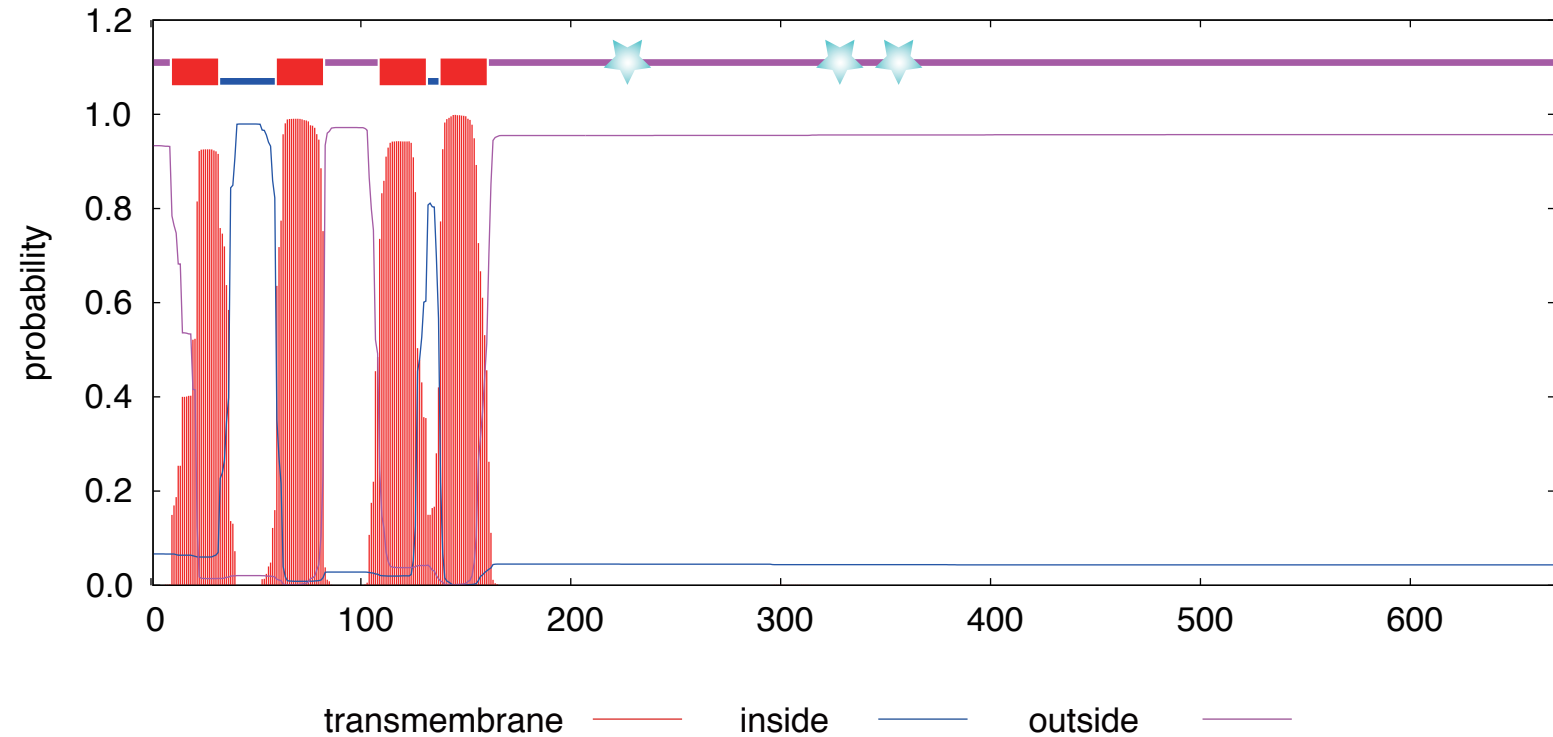

Supplement: Additional file 8: Figure S7. — The protein topology CNG channels involved in plant-pathogen interaction pathway. The stars denote nucleotide fixation in the protein. [file 12870_2015_463_MOESM8_ESM.pdf]
